# Unlocking the potential of rice bran protein: modification strategies and functional enhancements

**DOI:** 10.1016/j.crfs.2026.101335

**Published:** 2026-02-01

**Authors:** Jiao-jiao Yin, Yuan Zou, Kai Yao, Pan Gao, Wu Zhong, Xing-he Zhang, Li Wang

**Affiliations:** a Key Laboratory of Edible Oil Quality and Safety for State Market Regulation, Wuhan Polytechnic University, Wuhan, 430023, China; b Hubei Modern Agricultural Group Co., Ltd., Suizhou, 441300, China; c School of Modern Industry for Selenium Science and Engineering, Wuhan Polytechnic University, Wuhan, 430048, China

**Keywords:** Sustainable food ingredients, Physical modification, Chemical modification, Biological modification, Food application

## Abstract

Rice bran protein (RBP) is a high-quality plant protein with balanced amino acids and low allergenicity, but its industrial application is hindered by poor solubility and limited functionality. This review provides a critical analysis of modification strategies designed to overcome these challenges, categorizing them into physical, chemical, and biological approaches. Physical methods (e.g., ultrasound, high-pressure processing, extrusion) disrupt non-covalent interactions to improve solubility and interfacial properties. Chemical techniques, such as glycosylation and phosphorylation, enhance functionality through covalent conjugation, markedly improving emulsifying stability and solubility. Biological methods, including enzymatic hydrolysis and cross-linking, enable precise structural tailoring under mild conditions. A key contribution of this work is the introduction of a comparative, application-driven framework that systematically evaluates the efficacy, technological trade-offs, and optimal food applications of each method. The analysis highlights that the choice of modification must balance functional performance with considerations of scalability, cost, and clean-label requirements. Finally, future research priorities are identified, emphasizing the need for synergistic hybrid processes, performance validation in complex food matrices, and scalable process engineering. This comprehensive synthesis aims to guide the rational development of RBP as a sustainable, high-value functional ingredient for the food industry.

## Highlights

• Physical method improves solubility, suitable for beverages and drinks.
• Glycosylation enhances emulsification, ideal for sauces and creams.
• Enzymatic treatment boosts solubility & gelation, for plant-based foods.

## Graphical abstract

**Figure:**
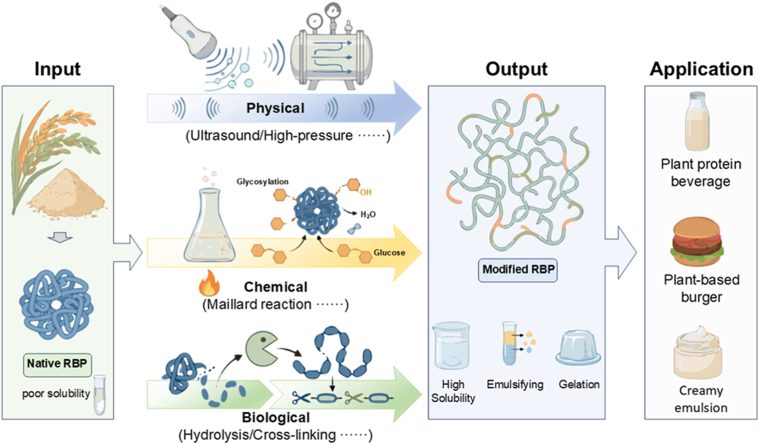

## 1 Introduction

With the continuous growth of the global population and increasing consumer demand for healthy and sustainable diets, the development of novel, high-quality plant-based protein resources has become a critical research direction in food science. Rice, as a major staple crop in the world, produces a large amount of rice bran as a byproduct, which is often underestimated in terms of its utilization value. Rice bran is a complex and nutrient-dense material, composed of fat (12%–23%), carbohydrates (34%–52%), protein (14%–16%), crude fiber (8%–10%), and a rich profile of bioactive compounds (Bollinedi et al., 2020; Eng and Mohd Rozalli, 2022). Among these, rice bran protein (RBP) exhibits a balanced amino acid profile, high biological value, and significantly lower allergenicity compared to soy and whey proteins, making it a highly promising and valuable plant-based protein source worthy of further development (Rivero Meza et al., 2024; Tang et al., 2022). Furthermore, compared to some legume proteins like pea protein that may exhibit lower solubility and limited emulsifying capacity in their native state, RBP possesses a distinct amino acid composition (e.g., higher sulfur-containing amino acids) that provides a favorable starting point for modification (Kumar et al., 2023). These comparative advantages in flavor, safety, and inherent functional potential underscore RBP's unique position as a sustainable and versatile protein ingredient for future food innovation.

RBP exhibits a biological value (∼72.6) that falls between those of casein (∼59.7) and whey protein (∼78.8) (Han et al., 2015). However, the presence of extensive hydrophobic regions and intermolecular disulfide bonds results in remarkably low solubility of native RBP, typically below 10% at acidic pH of 4–5, which directly limits its application in acidic food systems (Cho et al., 2022; Rivero Meza et al., 2024). Consequently, the poor solubility directly impairs its functional properties, including emulsification, foaming, and gelation capacity (Zolqadri et al., 2023). These limited processing functionalities significantly restrict the high-value application of RBP in liquid or semi-solid food systems, including beverages, emulsions, meat products, and gel-based foods. Consequently, RBP is predominantly utilized as animal feed or low-grade raw material, representing a substantial underutilization of a valuable resource.

To address this bottleneck, modifying RBP to enhance its functional properties has become an essential research direction. Currently, the primary modification approaches can be classified into three categories: physical, chemical, and biological methods (Akharume et al., 2021). Physical modification typically employs mechanical or thermal energy to alter the higher-order structure of proteins, instantaneously exposing functional groups (dos Santos et al., 2024; Venkateswara Rao et al., 2021). Chemical modification involves introducing or modifying charged chemical groups to change the electrostatic interactions of proteins (Wang et al., 2024; Zhang et al., 2024). Biological enzymatic modification, such as specific enzymatic hydrolysis and transglutaminase-mediated cross-linking, which is regarded as one of the most promising strategies due to its mild reaction conditions, high specificity, and superior safety (Liu et al., 2024; Singh et al., 2021). These methods act on RBP molecules through different mechanisms, aiming to improve their hydrophilic-lipophilic balance, molecular flexibility, and interfacial behavior, thereby comprehensively enhancing solubility, emulsifying stability, and gel strength. These modification strategies not only improve the application performance of RBP in food systems but also provide new insights for the advanced development of functional proteins.

To data, several reviews on RBP have been published, which can be broadly categorized into three types: (i) those summarizing modification approaches from a broad perspective applicable to plant proteins in general (Akharume et al., 2021; Dhiman et al., 2023; Venkateswara Rao et al., 2021), (ii) those focusing primarily on extraction techniques and general functional characterization of RBP (Huang et al., 2026; Phongthai et al., 2017; Rivero Meza et al., 2024), and (iii) those that, while touching upon the modification of RBP, remain largely descriptive, offering limited systematic analysis of the underlying mechanisms, structure-function relationships, or the suitability of different techniques for specific applications (Wang et al., 2021; Zolqadri et al., 2023). However, a critical gap remains: there is a lack of a dedicated, mechanistic review that systematically deconstructs how different modification strategies, physical, chemical, and biological, specifically target and ameliorate the inherent functional deficiencies of RBP, and subsequently links these structural changes to application-specific performance.

This review aims to fill this gap. The core objectives are to: (1) Elucidate the specific mechanisms by which each modification technology disrupts the restrictive native structure of RBP; (2) Critically compare the efficacy of these methods in enhancing key functionalities (solubility, emulsification, gelation) with supporting quantitative data where available; (3) Establish clear application guidelines by mapping the improved functional profile of modified RBP to specific food systems; and (4) Evaluate industrial scalability by balancing the functional benefits of each method against practical considerations of cost, scalability, safety, and clean-label compliance. By integrating mechanism, efficacy, and applicability, this work aims to serve as a foundational yet critical resource for the rational development of RBP as a high-value, functional food ingredient.

## 2 Physical modification

**Fig. 1:**
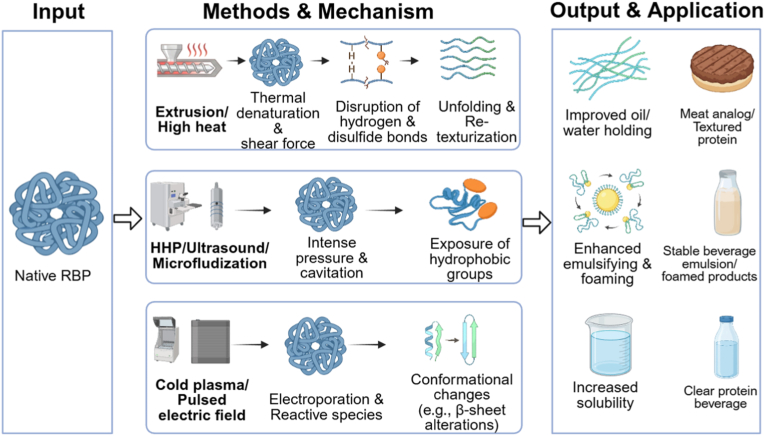
Physical modification of rice bran protein: disruption of non-covalent interactions for enhanced functionality.

Physical modification represents a fundamental approach to enhance the functionality of RBP by employing external energy inputs, including thermal, mechanical, and electromagnetic forces, to alter its higher-order structure without changing its primary chemical composition. This category of techniques primarily disrupts the non-covalent interactions (e.g., hydrogen bonds, hydrophobic associations, and ionic bonds) and, in some cases, disulfide linkages that stabilize the native protein conformation. The induced structural changes, such as molecular unfolding, aggregate dissociation, particle size reduction, and exposure of buried functional groups, collectively lead to significant improvements in key properties. These enhancements notably include increased solubility, enhanced emulsifying and foaming capacities, improved water- and oil-binding abilities, and modified gelation behavior. The physical modification methods and application of RBP are illustrated in Fig. 1.

### 2.1 Thermal processes

Thermal processing represents a significant modification approach that utilizes controlled thermal energy input to induce structural changes in proteins, thereby improving their functional properties. Depending on the heating principles and methods, it can be categorized into conventional thermal techniques and emerging electro-based technologies, as well as extended methods such as thermo-mechanical treatments (extrusion) and high-pressure thermo-explosive processes (steam flash explosion). All these methods fundamentally rely on thermal effects to achieve functional enhancement by modulating protein molecular conformation and aggregation behavior (Bi et al., 2024; Leal et al., 2021; Zolqadri et al., 2023).

#### 2.1.1 Traditional heat treatment

Traditional heat treatment relies on heating materials through external to internal heat conduction, convection, or radiation. During the heating process, polypeptide chains migrate in response to heat, leading to the disruption of hydrophobic and electrostatic interactions, hydrogen bonding, and disulfide bridges within proteins. The initial unfolding of the structure is mostly reversible, but with increasing temperature or prolonged time, proteins undergo irreversible denaturation, leading to the dissociation of secondary and tertiary structures, exposure of hydrophobic regions, and subsequent rearrangement and cross-linking through mechanisms such as hydrophobic interactions, hydrogen bonds, and disulfide bonds, ultimately leading to a newly formed, stable structural matrix (Akharume et al., 2021). This treatment markedly enhances the protein's solubility, emulsifying and gelling capabilities, as well as its water retention capacity, while concurrently diminishing the activity of anti-nutritional factors and improving overall digestibility.

In practical applications, the effectiveness of heat treatment is influenced by various operating parameters, including heating temperature, time, heating rate, ionic strength, and pH. By optimizing these conditions, targeted structural modification and functional enhancement of specific proteins can be achieved. Taking RBP as an example, studies have shown that dry heat pretreatment (120 °C, 60 min) can significantly enhance its oil-holding capacity (from 3.91 g/g to 4.31 g/g) and emulsifying activity (from 41.46% to 57.54%), but may slightly inhibit foam stability and solubility (Lv et al., 2017, 2018). This treatment not only can enhance select functional properties of RBP but also serves as a crucial pretreatment step for the bran itself. By inactivating endogenous lipases and lipoxygenases, it effectively suppresses lipid rancidity and extends the shelf life of rice bran prior to protein extraction, thereby ensuring the quality of the starting material (Lv et al., 2017, 2018). Therefore, heat treatment-based modification strategies can be tailored by adjusting process parameters according to the specific requirements of the final application, thereby expanding the application range of proteins in food systems.

The primary advantage of traditional heat treatment lies in its simplicity, scalability, and low cost, making it a mainstream industrial method for rice bran stabilization prior to protein extraction. However, its limitations for functional protein modification are notable: non-uniform heat transfer can lead to heterogeneous protein denaturation, and excessive temperatures or times readily promote irreversible aggregation, which may counteract desired solubility improvements. Its industrial relevance for RBP is therefore twofold: as a crucial pretreatment for bran stabilization and as a baseline thermal process, but it is often less precise for targeted functional enhancement compared to more advanced technologies.

#### 2.1.2 Microwave

Microwaves are a form of electromagnetic radiation operating within the frequency spectrum of 300 MHz to 300 GHz, can induce rapid heating of materials by causing intense movement of polar molecules (especially water molecules) through ion conduction and dipole rotation mechanisms (Zhou et al., 2024). Unlike traditional heating methods, microwave heating is a process where energy is transferred from the inside of the material to the surroundings, characterized by rapid heating and high thermal efficiency.

The efficacy of microwave treatment is governed by key parameters including microwave power (or power density), exposure time, sample moisture content, and the dielectric properties of the material. Compared to conventional heating, its advantages include rapid and volumetric heating, higher energy efficiency, and the potential for unique non-thermal effects. A significant limitation is the difficulty in achieving uniform heating in large or heterogeneous samples, and the capital cost of industrial-scale microwave systems can be high.

Microwave treatment generally reduces the α-helix and β-sheet content of RBP while increasing the proportion of β-turns and random coil structures. The molecular polarization induced by microwaves disrupts the non-covalent bonds that maintain the protein's spatial conformation, leading to partial unfolding of the protein structure and the exposure of hydrophobic groups and active sulfhydryl groups. This structural alteration enhances the interaction of RBP with water and oil, thereby improving both its solubility and emulsifying properties (Sun et al., 2025; Tian and Wang, 2015). Additionally, further structural modulation can be achieved through microwave plasma treatment. For instance, when applied at 160 W for 5 min, this technique facilitates the cleavage of hydrogen and disulfide bonds, promoting a transition from an ordered to a more disordered protein state. This process increases the surface exposure of hydrophobic amino acid residues and can generate carbonyl compounds and other oxidative products. The resulting enhancement in electrostatic repulsion between protein molecules, combined with greater surface hydrophobicity, collectively improves key functional attributes of RBP, including solubility, emulsifying and foaming capacities, as well as water- and oil-holding abilities (Liu et al., 2025). These findings underscore the potential of microwave-based technologies as efficient and controllable strategies for developing highly functional plant protein ingredients.

#### 2.1.3 Extrusion

Extrusion, as a continuous high-temperature short-time thermo-mechanical process, plays a pivotal role in modifying the structural and functional properties of RBP. By subjecting RBP to controlled combinations of heat, pressure, and shear forces within the extruder barrel, the native protein conformation is dynamically altered. This process induces partial denaturation and molecular unfolding, leading to the exposure of hydrophobic regions and reactive sulfhydryl groups. Subsequent reorganization under flow and pressure promotes the formation of new intermolecular interactions, predominantly through disulfide bonding and realigned non-covalent forces (Dhiman et al., 2023). Such structural rearrangement is fundamental to transforming RBP into a textured, fibrous matrix, making it particularly suitable for applications in plant-based meat analogues (Akharume et al., 2021).

Short-time, high-intensity extrusion conditions significantly influence the functional properties of plant proteins, including solubility, texture, emulsification, and gelation. The functional improvements conferred by extrusion on RBP are quantifiable and multifaceted. For instance, RBP processed under optimized conditions (130 °C, 20% moisture content) exhibited an 8.5% increase in oil-holding capacity, a 31.7% enhancement in emulsifying capacity, and a 38.2% improvement in foaming capacity compared with the non-extruded control, which can be attributed to the formation of a porous protein network (Leal et al., 2021). More notably, extrusion pretreatment at 140 °C with 25% moisture content led to RBP hydrolysates with significantly elevated emulsifying capacity (*P* < 0.05) relative to their native counterparts (Ouyang et al., 2024). These improvements underscore extrusion's utility in tailoring RBP for high-value applications, including structured foods, nutritional supplements, and clean-label protein ingredients.

The key advantages of extrusion for RBP modification include its continuous operation, high throughput, and unique ability to create fibrous, meat-like textures in a single step, which is directly relevant to the growing plant-based meat sector. However, limitations include the need for precise optimization of parameters (temperature, moisture, screw speed) to avoid excessive protein degradation or loss of nutritional quality. Challenges in scalability for some RBP feedstocks, economic viability at smaller scales, and a need for clearer structure-function-process models remain. Its industrial relevance is high, particularly for producing textured vegetable proteins, but successful application to RBP requires careful adaptation of process conditions specific to its composition.

#### 2.1.4 Steam flash explosion

Steam flash explosion (SFE) is an efficient and low-energy thermo-physical pretreatment technology widely employed in the food industry for the processing of fibrous or protein-rich biomass. The method involves exposing materials to pressurized saturated steam followed by instantaneous depressurization, which generates a synergistic effect combining high-temperature cooking and intense mechanical shearing (Rigueto et al., 2023). The efficacy of SFE is governed by several critical parameters, including steam pressure, temperature, residence time, and feedstock characteristics. In protein modification, excessive pressure or prolonged treatment can induce protein over-denaturation, amino acid degradation, and undesirable oxidative reactions, thereby impairing functional properties. Therefore, precise parameter control is essential to balance structural modification with the preservation of protein integrity.

The underlying mechanism of SFE involves the rapid release of kinetic energy during decompression, which disrupts the microstructure of protein matrices and promotes cell wall rupture. Concurrently, the high-temperature and high-pressure conditions induce protein conformational rearrangement, including the cleavage of disulfide bonds, molecular unfolding, and exposure of buried hydrophobic and sulfhydryl groups. These changes may also facilitate Maillard-type interactions between proteins and carbohydrates, altering molecular polarity and spatial conformation, which collectively contribute to modified functional behavior (Na et al., 2023).

In the context of RBP modification, SFE has been shown to significantly modulate physicochemical and functional attributes. For instance, treatment at 2.1 MPa for 210 s markedly reduced the surface hydrophobicity of RBP isolate from 137.5 to 17.5, while increasing its intrinsic viscosity (Na et al., 2023). This structural expansion and hydrophobic domain reorientation promote protein solubilization and enhance interfacial activity. Recent studies further demonstrate that SFE under optimized conditions (1.7 MPa/90 s) improves the solubility of RBP at pH 6.0 from 30.21% to 67.12%, attributed to increased surface hydrophilicity. Under 1.25 MPa/90 s, the emulsifying activity index and emulsifying stability index of RBP increased by 26.51% and 26.58%, respectively, due to enhanced exposure of hydrophobic groups and improved protein-solvent interactions (Bi et al., 2024).

The advantages of SFE include efficient disruption of lignocellulosic and protein matrices, leading to improved extractability and functionality, along with relatively low energy consumption compared to some mechanical methods. Its core limitations are the requirement for precise control of pressure and residence time to prevent over-processing, batch-mode operation which can limit throughput, and significant capital investment for pressure vessels. The industrial relevance of SFE for RBP modification is currently at the pilot-scale investigation stage. Its potential for commercial adoption hinges on demonstrating consistent functional benefits, economic feasibility in integrated biorefineries, and solving challenges related to the scalable handling of materials under explosive decompression.

### 2.2 Ultrasound

Ultrasonic treatment is a safe and efficient physical modification technique widely used in the modification of proteins. The underlying mechanism primarily relies on the cavitation effect: as ultrasound propagates through a liquid medium, it triggers the nucleation, rapid expansion, and implosive collapse of microcavities. This process generates localized extreme conditions, including temperatures near 5000 K, pressures around 50 MPa, along with intense shock waves and high-speed microjets (Guo et al., 2023). This phenomenon can disrupt non-covalent interactions, such as hydrogen bonds and hydrophobic associations, within protein molecules, resulting in alterations to their higher-order structures. These changes include a reduction in α-helix and β-sheet content, an elevation in random coil conformation, and the exposure of previously buried hydrophobic regions (Sun et al., 2021). Additionally, the mechanical shear force generated by ultrasound can break down protein particles into smaller nanoscale particles, improving their dispersibility, typically without disrupting the primary structure or molecular weight. It should be noted that under low power or prolonged treatment, protein re-aggregation may occur, forming larger aggregates. Furthermore, while ultrasonication is generally regarded as a non-thermal technology, the localized temperature increase resulting from cavitational collapse can still exert partial thermal influences on protein conformation and reactivity.

Ultrasonic treatment has demonstrated significant potential in modifying the functional properties of RBP. Under optimized conditions, such as at 200 W, ultrasonication disrupts the non-covalent interactions that stabilize the spatial structure of RBP, leading to partial unfolding, reduction in particle size, and improvements in water-holding capacity, oil-holding capacity, and emulsifying activity (*P* < 0.05) (Guo et al., 2023). It is noteworthy that although performance declines when power exceeds 200 W, the functional attributes remain markedly superior to those of untreated RBP. The mechanism underlying these enhancements involves the exposure of hydrophobic groups and an increase in surface hydrophobicity, which collectively contribute to improved emulsion stability. For instance, when applied to emulsions prepared from RBP and chlorogenic acid, ultrasound treatment reduced droplet size, promoted uniform distribution, increased viscosity, and minimized droplet aggregation and phase separation during storage, thereby significantly enhancing emulsion stability (Wang et al., 2022).

Further structural analyses indicate that low-frequency ultrasound (20 kHz) can modify the secondary structure of RBP concentrate, characterized by an increase in β-sheet and random coil contents alongside a decrease in α-helix and β-turn proportions. These conformational changes, coupled with a reduction in particle size, are associated with enhanced foaming and oil-binding capacities (Sharma et al., 2023). However, the efficacy of ultrasonic modification is highly dependent on processing parameters. Excessive power or prolonged treatment may lead to protein over-aggregation or structural damage, resulting in diminished emulsifying properties (Sun et al., 2021). Therefore, precise optimization of ultrasonic conditions, including power, duration, and frequency, is essential to achieve targeted functional improvements in RBP. As a non-thermal, energy-efficient technology, ultrasound represents a promising green processing strategy for enhancing the functionality of plant-derived proteins in sustainable food applications.

### 2.3 Pulsed electric field

Pulsed electric field (PEF) processing is a non-thermal technology that subjects materials to short, high-intensity electric field pulses (typically 0.1–80 kV/cm) at ambient temperature. Its fundamental mechanism involves electroporation, wherein the induced transmembrane potential exceeds a critical threshold, leading to the formation of reversible or irreversible pores in cellular or macromolecular structures, thereby significantly enhancing membrane permeability (Chakraborty et al., 2025; Thongkong et al., 2023).

In the modification of RBP, PEF has been shown to induce conformational changes, notably promoting the conversion of β-turns to β-sheets. These structural transformations are associated with measurable improvements in functional properties. For instance, studies report increases in oil-holding capacity by 20.29–22.64%, enhancement of emulsifying properties by 3.3–12.0% (*P* < 0.05), and significant elevation in foaming capacity and foam stability, reaching 1.8- to 2.9-fold that of untreated RBP (Thongkong et al., 2023). Such modifications highlight the potential of PEF as an efficient, green physical method for enhancing the functionality of plant proteins.

Despite these promising results, the practical application of PEF in RBP modification faces several challenges. Key among these is the need for precise control over process parameters, such as electric field strength, pulse number, specific energy input, and treatment temperature, to avoid over-processing, which may lead to protein aggregation or loss of native functionality. Additionally, the scalability of PEF technology, equipment costs, and energy efficiency in continuous industrial operations require further optimization. A deeper understanding of the long-term stability, nutritional integrity, and sensory compatibility of PEF-modified RBP in complex food matrices also remains essential for its successful commercial adoption.

### 2.4 High hydrostatic pressure

High Hydrostatic Pressure (HHP) processing serves as a prominent non-thermal technology extensively applied for the functional modulation of plant-based proteins. This treatment involves applying high static pressure (typically 100–600 MPa) in conjunction with suitable temperature, time, and solution conditions (such as pH and ion strength) to induce changes in protein conformation, thereby regulating its functional properties. The mechanism of action of HHP is primarily based on Le Chatelier's principle, which promotes molecular interactions by reducing system volume, leading to reversible or irreversible denaturation, unfolding, or aggregation of proteins. It is worth noting that HHP mainly affects non-covalent bonds (such as hydrogen bonds, ionic bonds, and hydrophobic interactions) that maintain the higher-order structure of proteins, without disrupting covalent bonds or primary structure, and without causing amino acid degradation, thus preserving the nutritional value of food well (Yi and Liu, 2023).

Research has shown that HHP treatment can significantly enhance protein solubility. Yi and Liu (2023) conducted HHP treatment on RBP and found that at 200 MPa for 40 min, the solubility of RBP increased by 76.69% compared to its initial untreated state at pH 7.0. This effect is likely due to the pressure-induced unfolding of protein structures, which cleaves linkages within and between polypeptide chains, disrupts compact molecular arrangements, and promotes enhanced protein-water interactions, ultimately leading to improved solubility (Barbhuiya et al., 2021; Yi and Liu, 2023). Additionally, HHP can serve as an efficient pretreatment method to assist enzymatic modification. Studies have shown that pretreating RBP at 200 MPa for 30 min, followed by limited enzymatic hydrolysis with pancreatin, can effectively reduce interfacial tension, promote uniform dispersion and stability of oil droplets, and significantly improve its emulsifying properties (Wang et al., 2025). This result indicates that the combined use of HHP and enzymatic methods can synergistically enhance the functional performance of RBP, thereby offering a theoretical foundation and technical framework for its utilization as a plant-based protein emulsifier in beverage and food applications. However, high equipment costs and limited scalability for continuous processing are critical considerations for the practical application of HHP technology.

### 2.5 Cold plasma technology

Cold plasma, an emerging non-thermal technology, enables the surface modification of proteins through exposure to a reactive gas medium energized by electrical or electromagnetic discharge. This process generates a mixture of reactive nitrogen and oxygen species (RNS/ROS) along with ultraviolet radiation, which collectively interact with protein structures. The reactive species facilitate the cleavage of covalent bonds, including the oxidation of sulfur-containing amino acids and rupture of disulfide linkages, leading to conformational changes and structural rearrangement in proteins (Akharume et al., 2021; Dhiman et al., 2023). The main advantage of this technology is its ability to perform dry, non-thermal surface modification at near-ambient temperatures, making it suitable for heat-sensitive materials.

In the modification of RBP, cold plasma treatment has been shown to induce controlled structural unfolding and functional enhancement. Studies indicate that moderate treatment durations (4–6 min) effectively disrupt disulfide bonds, reduce α-helix and β-sheet content by 17.24% and 31.21%, respectively, and increase the proportion of β-turns and random coils. These structural changes enhance molecular flexibility at oil-water interfaces, thereby significantly improving emulsifying capacity, from below 20–55.03 m^2^/g (Li et al., 2026).

However, the efficacy of cold plasma is highly dependent on precise control of treatment parameters such as voltage, gas composition, exposure time, and sample characteristics. Prolonged treatment beyond an optimal window can lead to excessive oxidation, protein reaggregation, and diminished functional performance. These sensitivity to processing conditions, along with challenges in scaling the technology for continuous industrial application, currently limits its widespread adoption in food protein modification.

### 2.6 Microfluidization

Microfluidization, also termed dynamic high-pressure microfluidization (DHPM), is an advanced homogenization technology that applies significantly higher pressures and collision energies than conventional high-pressure homogenization. During processing, fluid is forced through precisely designed microchannels, subjecting the material to extreme shear, cavitation, high-frequency impact, and rapid pressure fluctuations, which collectively induce substantial changes in molecular structure and functional properties (de Carvalho et al., 2024; Kavinila et al., 2023). A major advantage of microfluidization over conventional homogenization is its ability to generate more uniform and stable emulsions or dispersions with smaller particle sizes due to the combined shear, impact, and cavitation forces.

The modification mechanism primarily involves the disruption of non-covalent interactions within and between protein molecules. The intense hydrodynamic forces dissociate protein aggregates, reduce particle size, and increase surface area, while also partially unfolding protein structures to expose buried hydrophobic and sulfhydryl groups. This structural rearrangement enhances protein–solvent interactions, improves solubility, and modifies interfacial behavior, thereby boosting emulsifying and foaming properties (de Carvalho et al., 2024; Diana Kerezsi et al., 2024; Li et al., 2025; Zhao et al., 2024).

In the context of RBP, DHPM has been employed to prepare RBP-gum arabic (GA) stabilized oil-in-water emulsions. Under optimized conditions (120 MPa), the treatment significantly enhances emulsifying activity index and emulsifying stability index, reaching up to 118.70 m^2^/g and 112.75 min, respectively. This improvement is attributed to the exposure of charged, hydrophobic, and hydrophilic groups on RBP, which strengthens interactions at the oil-water interface. Additionally, the intense shear forces reduce droplet size and improve dispersion uniformity, while increased droplet number and entanglement under pressure contribute to higher apparent viscosity (Yu et al., 2025). However, the functional benefits are highly pressure-dependent. Excessive treatment (e.g., >160 MPa) can lead to over-shearing, disruption of the interfacial film, droplet coalescence, and protein reaggregation via disulfide bonding, ultimately reducing emulsifying performance and apparent viscosity (Yu et al., 2025). These findings highlight the importance of optimizing processing parameters, including pressure, pass number, and protein concentration, to balance structural modification with functional enhancement. The scalability of DHPM for continuous industrial operation, along with energy consumption and equipment durability, remain practical challenges that require further evaluation for its widespread adoption in RBP modification.

## 3 Chemical modification

**Fig. 2:**
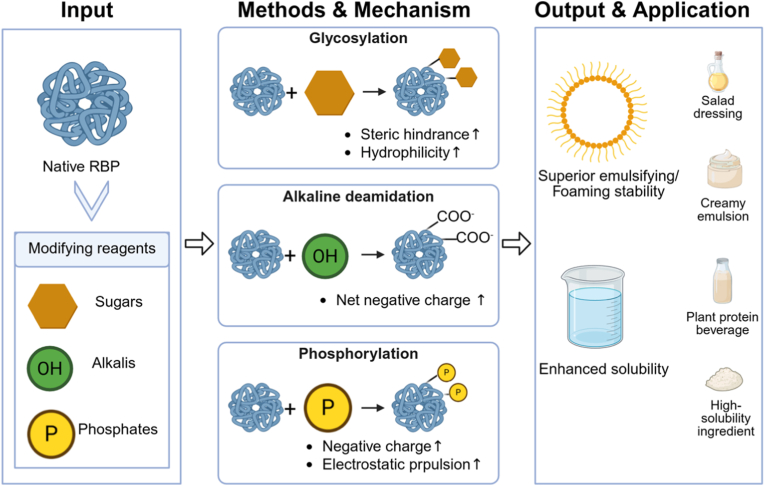
Chemical modification of rice bran protein: covalent conjugation for improved hydrophilicity and stability.

Chemical modification involves the reaction of proteins with specific reagents to cleave or form new bonds, thereby altering the native protein architecture. This approach primarily exploits the reactivity of side-chain functional groups, including amino, carboxyl, disulfide/thiol, etc. To modulate protein biophysical characteristics and functionality. A key objective is to modify the net charge of proteins through substitution of amino or hydroxyl groups on specific amino acid residues. Studies indicate that chemically modified proteins often demonstrate enhanced functional performance compared to their native counterparts. However, widespread commercialization of these techniques remains constrained by challenges such as the generation of toxic by-products, high operational costs, consumer acceptance issues, and regulatory limitations (Zhang et al., 2018). The chemical modification methods and application of RBP are illustrated in Fig. 2.

### 3.1 Chemical glycosylation

Glycosylation is a biochemical process that involves the covalent attachment of carbohydrate groups to specific sites on proteins, such as the N-terminus of the polypeptide chain or the side-chain functional groups of certain amino acid residues. The Maillard reaction is a typical non-enzymatic glycosylation method, which is a heat-catalyzed covalent grafting reaction between amino and carbonyl groups. Protein glycosylation fundamentally represents the initial phase of the Maillard reaction. As such, the native conformation of the protein remains largely unaltered, preserving its original functional characteristics. Following relatively straightforward conjugation with polysaccharides, glycosylated proteins not only maintain their inherent functional attributes but also exhibit enhanced performance due to the contribution of the polysaccharide moieties. Studies have demonstrated that glycosylation modification can effectively improve key functional properties of proteins, including solubility, emulsifying and foaming capabilities, as well as thermal stability (Cheng et al., 2022; Karbasi and Askari, 2021; Zhang et al., 2018; Zhao et al., 2022). The efficiency and outcomes of protein glycosylation are influenced by multiple factors, including reaction temperature and duration, pH, relative humidity, the structural characteristics of both the polysaccharide and protein components, their mass ratio, and the degree of glycosylation achieved (Akharume et al., 2021; Zhang et al., 2018). Furthermore, no harmful or toxic compounds have been observed during the formation of protein-polysaccharide complexes, making it considered a safe processing method for protein modification (Zhang et al., 2018).

The glycosylation of RBP with maltose, as reported by Sun et al. (2025), led to significant changes in its functional profile. Following modification, RBP exhibited markedly enhanced solubility, particularly under pH conditions above 6.0. Concurrently, emulsifying activity increased from 34.82 to 46.24 m^2^/g, and foaming capacity rose from 8.66% to 24.51%. These results suggest that glycosylation effectively improves surface-active and solubility-related properties (Sun et al., 2025). Additionally, the Maillard reaction-induced modification of rice bran globulin (RBG) significantly enhances its functional properties. Following glycation, RBG exhibits notable improvements in solubility (increasing from 17.47% to 37.23%), emulsifying activity (from 0.17 to 0.31), emulsifying stability (from 50.66% to 65.37%), as well as foaming capacity and foam stability, which increased by 1.26- and 1.12-fold, respectively (Zhang et al., 2024). These functional enhancements are primarily attributed to structural and interfacial modifications mediated by glycosylation. On one hand, the covalent grafting of saccharide molecules, such as chitooligosaccharide, introduces numerous hydrophilic groups (e.g., -OH, -NH_2_) onto RBG, while also promoting partial protein unfolding and exposure of internal hydrophilic regions. These changes collectively reduce the surface hydrophobicity of the protein, strengthen its interaction with water, and substantially improve solubility. On the other hand, the attached carbohydrate chains not only enhance hydrophilicity but also provide steric hindrance, effectively shielding hydrophobic patches from direct contact with the oil phase and thereby inhibiting droplet coalescence, which contributes to improved emulsifying activity and stability. Furthermore, glycation increases protein structural flexibility and promotes a more expanded conformation, enabling faster adsorption, spreading, and formation of a stable interfacial film at the air-water interface, which in turn enhances foaming capacity and foam stability (Zhang et al., 2024).

Despite these advantages, the Maillard reaction requires careful control to avoid advanced glycation stages that may lead to browning, off-flavors, or loss of nutritional quality. Future efforts should focus on optimizing reaction conditions for RBP, exploring alternative carbohydrate sources with tailored structural features, and evaluating the nutritional and sensory impacts of glycosylated RBP in real food systems to facilitate its industrial adoption.

### 3.2 Deamidation

Chemical deamidation is a process that hydrolyzes the amide groups of asparagine and glutamine residues in proteins under acidic or alkaline conditions at elevated temperatures, converting them into carboxyl groups with the release of ammonia. This modification increases the net negative charge on the protein surface, enhances electrostatic repulsion between molecules, reduces aggregation tendency, and promotes structural unfolding, thereby exposing internal hydrophobic and hydrophilic regions. These changes improve molecular flexibility and surface hydrophilicity, collectively contributing to enhanced functional properties such as solubility and emulsifying capacity (Akharume et al., 2021; Castillo-Ortega et al., 2024; Chen et al., 2021). The extent of deamidation is controlled by key parameters: pH (acidic or alkaline), temperature, reaction time, and the ionic strength of the medium. Alkaline deamidation is generally faster but carries a higher risk of side reactions. The primary advantage of chemical deamidation is its effectiveness in dramatically improving protein solubility and emulsifying properties. Its industrial relevance has been established for certain proteins like wheat gluten, but application to RBP requires optimization to balance functionality gains against potential formation of undesired compounds like lysinoalanine under harsh conditions.

In the modification of RBP, controlled alkaline deamidation, using mild agents such as NaHCO_3_ or NaOH, has been shown to significantly enhance solubility without substantial peptide bond cleavage or molecular weight reduction. Optimal modification is achieved under carefully regulated condition treatment at pH 8 and 100 °C for 30 min yields moderately soluble deamidated RBP (∼40%) with minimal side reactions (Guan et al., 2017). More severe conditions, such as pH 12 and 120 °C for 15–30 min, can further increase solubility to approximately 90%, but often induce undesirable reactions including amino acid racemization and lysinoalanine formation, which may compromise nutritional quality and safety (Guan et al., 2017). Therefore, industrial applications typically employ milder alkaline protocols to balance functionality with protein integrity.

Recent advances have focused on developing greener and safer deamidation methods. For instance, natural deep eutectic solvents composed of glucose and citric acid have demonstrated effectiveness in enhancing the solubility and functionality of proteins, offering a more sustainable alternative to conventional chemical treatments (Shahid et al., 2024). These emerging approaches align with the growing demand for environmentally benign and nutritionally conscious protein modification strategies in the food industry.

### 3.3 Phosphorylation

Chemical phosphorylation entails the covalent bonding of a phosphate group (PO_3_) to nucleophilic amino acid residues, such as serine, threonine, tyrosine, lysine, arginine, or cysteine, via their reactive side chains (-OH, -NH_2_, or -SH groups). The degree of phosphorylation is influenced by factors including protein type, phosphorylating agent, and reaction conditions. Commonly used phosphorylating reagents include sodium tripolyphosphate (STP), sodium trimetaphosphate (STMP), and phosphorus oxychloride (POCl_3_). Notably, the U.S. Food and Drug Administration classifies STP, STMP, and POCl_3_ as Generally Recognized as Safe (GRAS) substances when utilized in accordance with good manufacturing practices (Akharume et al., 2021). Phosphate bonds exhibit stability across a broad temperature range (up to 120 °C) and pH conditions (2.0–10.0), rendering phosphorylated proteins highly suitable for food processing applications. The incorporation of phosphoryl groups enhances protein hydrophilicity by promoting deprotonation, thereby increasing the net negative surface charge and consequently improving solubility.

By using sodium trimetaphosphate (STMP) to phosphorylate RBP across a range of pH values (3, 5, 7, 9, 11), it was demonstrated that phosphorylation effectively enhances RBP functionality. Under alkaline conditions (pH 9.0), phosphorylation successfully introduced phosphate groups, increasing the protein surface negative charge and enhancing electrostatic repulsion. These modifications led to a significant improvement in solubility (from 6.67% to 58.4%) and emulsifying activity, with the emulsifying activity index rising from 1.70 m^2^/g to 13.72 m^2^/g. Structural analysis revealed that phosphorylation induced a shift in protein secondary structure: β-sheet content decreased, while α-helix and β-turn contents increased, indicating partial protein unfolding, exposure of hydrophobic regions, and enhanced molecular flexibility. These structural changes collectively improved the protein's adsorption and stabilization capabilities at interfaces. Thus, phosphorylation represents an effective strategy for synergistically enhancing RBP functionality through charge modification and conformational regulation, providing a theoretical foundation for its application in food emulsion systems (Hu et al., 2019; Si-Kyung Lee et al., 2004). Moreover, phosphorylation with sodium tripolyphosphate modified the structural characteristics of rice RBG, transforming its microstructure from spherical particles into irregular flakes and markedly increasing surface hydrophobicity. These changes provide a theoretical foundation for further development and utilization of RBG in functional applications (Ma et al., 2017).

These research findings indicate that phosphorylation treatment can enhance the functionality and application properties of RBP, offering potential opportunities for its use in the food industry. However, its practical industrial implementation still faces several challenges. The process requires precise control over reaction parameters such as pH, temperature, and reagent concentration to avoid over-modification or undesirable side reactions. The scalability of phosphorylation under consistent and economically viable conditions remains a concern, particularly regarding reagent cost, energy consumption, and the management of chemical residues. Furthermore, regulatory approval for phosphorylated proteins in food products must address safety and labeling requirements, which could limit its widespread adoption. Future research should focus on optimizing process efficiency, evaluating environmental impacts, and validating the functional performance of phosphorylated RBP in complex food matrices under industrial-scale conditions.

## 4 Biological modification

**Fig. 3:**
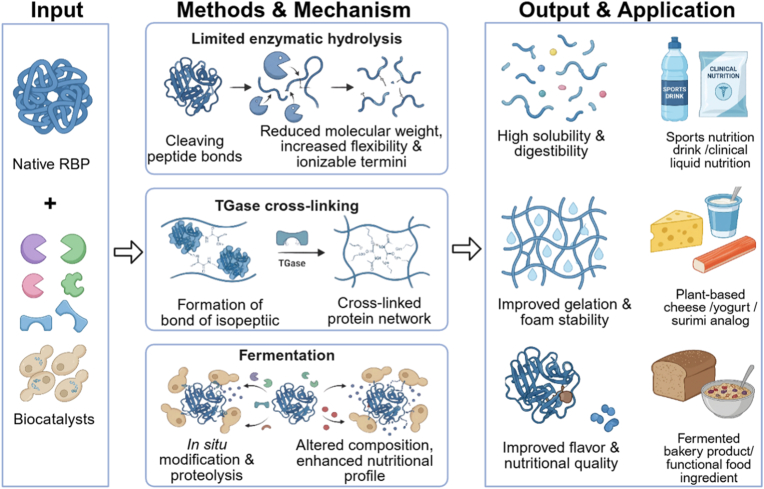
Biological modification of rice bran protein: enzymatic and microbial tailoring for specific structures.

Compared to chemical approaches, enzymatic modification operates under milder conditions, exhibits higher specificity, achieves faster reaction kinetics, and avoidance of harmful chemical reagents, making it more in line with the food industry's requirements for safety and controllability. Therefore, enzyme modification of plant proteins demonstrates significant research potential and application value. How to achieve modified proteins with desired functional properties by precisely controlling reaction conditions has become a key focus in this field (Singh et al., 2021; Y. Wang et al., 2025). The biological modification methods and application of RBP are illustrated in Fig. 3.

### 4.1 Enzymatic hydrolysis

Enzymatic hydrolysis of proteins involves the cleavage of peptide bonds by proteases under controlled conditions. The efficiency and outcome of this process are influenced by multiple key parameters, including enzyme type and specificity, enzyme-to-substrate ratio, temperature, pH, reaction time, and the degree of hydrolysis (DH). The primary advantages of enzymatic modification are its high specificity, mild reaction conditions, and the clean-label nature of the process. (Akharume et al., 2021; Y. Wang et al., 2025).

During hydrolysis, the higher-order structure of proteins is altered, often exposing buried hydrophobic regions. Limited hydrolysis (degree of hydrolysis, DH < 10%) has been shown to improve functional properties such as solubility, emulsifying capacity, and foaming ability, while avoiding the bitterness associated with extensive hydrolysis. The reduction in peptide size, increased solvent accessibility, and elevation of ionizable groups and net surface charge contribute to these enhancements. For instance, trypsin-mediated limited hydrolysis of RBP at DH levels of 1%, 3%, and 6% reduces high-molecular-weight aggregates, increases low-molecular-weight peptides, improves molecular flexibility, and elevates solubility (Zang et al., 2019). Conversely, excessive hydrolysis (DH > 10%) often leads to the accumulation of hydrophobic residues, imparting bitterness, and may result in the loss of functional activity (Singh et al., 2019; Y. Wang et al., 2025). When papain was used to hydrolyze RBP for different durations (30, 60, and 150 min, corresponding to DH values of 15%, 25%, and 32%), the β-sheet and β-turn contents decreased, while α-helix and random-coil contents increased, indicating a shift from ordered to disordered conformations. Although solubility, emulsifying, and foaming properties initially improved, further increases in DH led to declines in emulsifying and foaming performance as well as thermal stability (Singh et al., 2021). Therefore, enzymatic hydrolysis serves as a targeted tool for modifying protein functionality in the food industry, but its success relies on precise control of the hydrolysis degree and process conditions to balance functional enhancement with potential drawbacks such as bitterness and structural over-degradation.

### 4.2 Enzymatic cross-linking

Enzyme crosslinking aims to build a stronger protein network structure by introducing covalent bonds between peptide chains, thereby improving its functional properties (Y. Wang et al., 2025). Transglutaminase (TGase, EC 2.3.2.13) is currently the only commercially available food-grade crosslinking enzyme. It has been extensively documented to improve protein texture, viscosity, foaming properties, and surface hydrophobicity (Akharume et al., 2021). Its catalytic mechanism involves facilitating acyl transfer reactions between glutamine (acyl donor) and lysine (acyl acceptor) residues, resulting in the formation of intra- or intermolecular crosslinks and promoting protein polymerization. The cross-linking efficiency is governed by key parameters such as enzyme concentration (activity), incubation temperature and time, pH (optimal for TGase activity), and substrate (protein) concentration. The main advantage of enzymatic cross-linking, particularly with TGase, is its ability to naturally strengthen protein networks without introducing foreign chemicals, thereby improving gel strength and texture in a label-friendly manner.

In the modification of RBP, TGase crosslinking has been shown to effectively alter the physicochemical properties of RBP particles and their adsorption behavior at interfaces. For instance, when RBP was treated with TGase at 0.5 kat/kg for 3 h, its foam capacity increased to 375% and foam volume ratio reached 79.45%. The crosslinked RBP particles adopted a more rigid conformation, which contributed to excellent foam stability. The enzyme-induced crosslinking also elevated the interfacial elastic modulus, thereby reinforcing the stability of the foam system (Wu et al., 2023). Furthermore, TGase has been employed to fabricate novel RBP-based emulsion gels. The enzyme promotes intermolecular crosslinking among RBP molecules, modifies protein secondary structure, and facilitates the formation of a more homogeneous and compact three-dimensional network. These structural improvements significantly enhance the rheological and textural properties of the gel. The crosslinking process also increases the adsorption capacity of RBP at the oil–water interface and promotes protein aggregation, further strengthening the stability of the emulsion gel system (Liu et al., 2024). Notably, the resulting gel exhibits effective encapsulation of curcumin, highlighting its potential as a delivery vehicle for bioactive compounds. This approach offers a promising strategy for the stabilization and functional enhancement of protein-based matrices in food applications (Liu et al., 2024).

### 4.3 Fermentation

Beyond traditional physical and chemical methods, fermentation offers a unique biological route to modify RBP by harnessing the action of endogenous proteases. Fermentation is a controlled application that utilizes the growth and metabolism of beneficial microorganisms to improve the bioavailability of health-promoting bioactive substances and reduce unwanted anti-nutrients or harmful substances in food raw materials, thereby transforming food raw materials into useful products. Following fermentation, the nutritional profile of rice bran is enhanced, exhibiting elevated levels of protein, carbohydrates, minerals, and specific fatty acids. The resulting fermented product can function as a high-quality additive in animal feed formulations (Manlapig and Matsui, 2025; Omarini et al., 2019; Xie et al., 2024). The success of fermentation in modifying RBP depends on critical parameters including the type and strain of microorganisms, fermentation time and temperature, substrate composition (e.g., moisture, nutrient availability), and whether the process is solid-state or submerged.

Researchers have employed yeast and natural fermentation to modify RBP. The results demonstrate that fermentation treatment significantly enhanced the physicochemical and functional properties of RBP. The microbial enzymatic activity during fermentation promoted protein hydrolysis, generating new peptides and amino acids, which increased the content of both polar amino acids (e.g., serine, threonine) and non-polar amino acids (e.g., alanine, valine), thereby improving the hydrophilicity and hydrophobicity of the protein. Concurrently, fermentation induced an increase in the proportion of β-sheet structures within the protein conformation, contributing to improved thermal stability (denaturation temperature of yeast-fermented-RBP-concentrate reached 108.01 °C). Furthermore, fermentation reduced the insolubility near the protein's isoelectric point and enhanced solubility, foaming capacity, and emulsifying properties under alkaline pH conditions. These modifications render the fermented RBP more suitable for applications in food systems such as bakery products (Chinma et al., 2014).

Fermentation can significantly improve the solubility, functional properties, and antioxidant activity of RBP, while also enhancing its thermal stability. The process is environmentally friendly, cost-effective, and suitable for food industry applications. However, fermentation is less controllable, prone to fluctuations in microbial strains and conditions, which may lead to inconsistent product quality between batches. Additionally, the relatively long fermentation time limits production efficiency.

## 5 Comparative analysis and application for modified RBP

A synthesis of the research on physical, chemical, and biological modification strategies reveals distinct pathways for regulating the structure and functionality of RBP, governed by their unique mechanisms of action. As systematically compared in Table 1, the efficacy of each method in quantitatively improving key RBP properties, alongside its inherent advantages, limitations, and most suitable food application scenarios, provides a critical foundation for targeted technology selection.

**Table 1:** A comprehensive and critical comparison of modification strategies for rice bran protein.

Category	Method (Key Conditions)	Primary Mechanism	Key Improvement in RBP Properties	Advantages	Limitations/Challenges	Recommended Primary Applications	References
Physical	Traditional Heat Treatment (Dry heat, 120 °C, 60 min)	Thermal denaturation disrupts hydrophobic interactions, hydrogen bonds, and disulfide bonds, leading to unfolding and re-aggregation.	• Oil-holding capacity: ↑ from 3.91 to 4.31 g/g • Emulsifying activity: ↑ from 41.46% to 57.54 • Water-holding capacity: ↑ • *Note: May inhibit foam stability and solubility*	Low cost, scalable, effective for bran stabilization (inactivates lipases).	Non-uniform heating; risk of excessive aggregation; functional improvements can be inconsistent.	Stabilized rice bran as raw material; protein ingredients for baked goods where emulsifying/oil-binding is key.	Lv et al. (2018); Lv et al. (2017)
	Microwave & Microwave Plasma (160 W, 5 min)	Ionic conduction/dipole rotation generate heat; non-thermal electric field effects and plasma reactive species disrupt S-S/H-bonds.	• Solubility, emulsifying, foaming, water/oil-binding capacities: ↑ • Secondary structure: α-helix/β-sheet ↓; random coil ↑	Rapid, volumetric heating; can induce significant structural disordering.	Requires precise control; potential for oxidative damage; equipment specialization.	High-functionality protein ingredients for emulsions and foamed products.	Liu et al. (2025); Tian and Wang (2015)
	Extrusion (130–140 °C, 20%–25% moisture)	Combined thermal, shear, and pressure forces cause denaturation, unfolding, and re-texturization via disulfide bonding.	• Oil-holding capacity: ↑ 8.5% • Emulsifying capacity: ↑ 31.7% • Foaming capacity: ↑ 38.2% • Hydrolysate functionality: ↑	Continuous process; creates fibrous texture ideal for meat analogues.	High shear/temperature may degrade protein; requires precise moisture/temperature control.	Textured plant-based proteins (meat analogues); protein-rich snacks.	Leal et al. (2021); Ouyang et al. (2024)
	Steam Flash Explosion (1.25–1.7 MPa, 90 s)	Thermo-mechanical shock from instant pressure release disrupts cell walls, depolymerizes proteins, and exposes hydrophobic groups.	• Solubility (pH 6): ↑ from 30.21% to 67.12% • EAI: ↑ 26.51% • ESI: ↑ 26.58% • Surface hydrophobicity: ↓ from 137.5 to 17.5	Efficient biomass/aggregate disruption; significantly enhances solubility and emulsibility.	High equipment cost; risk of over-processing and protein degradation; batch operation.	Soluble protein concentrates for neutral-pH beverages and emulsified food systems.	Bi et al., 2024; Na et al. (2023)
	Ultrasound (200 W, 20 kHz)	Cavitation-induced shear forces and micro-jets disrupt non-covalent bonds, reduce particle size, and expose hydrophobic groups.	• Emulsifying activity: ↑ (*P* < 0.05) • Foaming capacity & oil-binding: ↑ • Secondary structure: β-sheet & random coil ↑; α-helix & β-turn ↓.	Green, non-thermal technology; improves solubility and interfacial properties effectively.	Risk of over-aggregation with excessive power/duration; scale-up for continuous flow is challenging.	Emulsion stabilizers (e.g., in RBP-chlorogenic acid systems); foaming agents.	Guo et al. (2023); Sharma et al. (2023); Sun et al. (2021)
	Pulsed Electric Field	Electroporation alters membrane/protein structure, inducing conformational changes (e.g., β-turn to β-sheet conversion).	• Oil-holding capacity: ↑ 20.29–22.64 • Emulsifying activity: ↑ 3.3–12.0% (*P* < 0.05) • Foaming capacity & stability: ↑ 1.8–2.9 fold	Non-thermal, energy-efficient; can improve multiple functionalities simultaneously.	Requires precise control of field strength and pulses; industrial application for protein modification is nascent.	Protein ingredients with enhanced oil-binding, emulsifying, and foaming properties.	Thongkong et al. (2023)
	High Hydrostatic Pressure (200 MPa, 30–40 min)	Pressure-induced disruption of non-covalent bonds (hydrogen, hydrophobic, ionic) leads to protein unfolding and solubilization.	• Solubility (pH 7): ↑ 76.69% • Emulsifying properties: ↑ (synergistic with enzymatic hydrolysis)	Excellent clean-label profile; preserves nutrition; very effective for solubility enhancement.	Very high capital investment; batch processing; limited scalability for continuous production.	Premium clear protein beverages; high-value ingredients requiring exceptional solubility.	Wang et al. (2025); Yi and Liu (2023)
	Cold Plasma (4–6 min treatment)	Reactive oxygen/nitrogen species (RONS) oxidize sulfur-containing amino acids and disrupt disulfide bonds, modifying surface properties.	• Emulsifying capacity: ↑ to 55.03 m^2^/g • Secondary structure: α-helix ↓ 17.24%, β-sheet ↓ 31.21%	Non-thermal surface modification; can significantly improve interfacial activity.	Highly sensitive to process parameters; risk of over-oxidation; scaling technology is complex.	Protein-based whipped creams; emulsion stabilizers requiring improved surface activity.	Li et al. (2026)
	Microfluidization (120 MPa)	Intense shear, cavitation, and impact forces dissociate aggregates, reduce droplet/particle size, and enhance interfacial adsorption.	• EAI: ↑ to 118.70 m^2^/g • ESI: ↑ to 112.75 min • Emulsion viscosity: ↑	Can produce extremely stable emulsions with small droplet size; improves texture.	Excessive pressure (>160 MPa) can break emulsion films; high energy consumption per unit.	High-stability O/W emulsions (e.g., RBP-Gum Arabic systems); creamy formulations.	Yu et al. (2025)
Chemical	Glycosylation (Maillard)	Covalent conjugation between protein amino groups and reducing sugar carbonyls, increasing hydrophilicity and steric hindrance.	• Solubility (RBG): ↑ from 17.47% to 37.23% • EAI (RBG): ↑ from 0.17 to 0.31 • ESI (RBG): ↑ from 50.66% to 65.37% • Foaming capacity/stability: ↑ 1.26/1.12 fold	Uses GRAS reactants; dramatically improves emulsifying and foaming stability.	Requires controlled conditions to prevent advanced browning/off-flavors; reaction efficiency varies.	Salad dressings, sauces, plant-based creams demanding high emulsion/foam stability.	Zhang et al. (2024); Sun et al. (2025)
	Alkaline Deamidation (pH 8, 100 °C, 30 min)	Hydrolysis of Asn/Gln side-chain amides to carboxylates, increasing net negative charge and electrostatic repulsion.	• Solubility: ↑ to ∼40% (mild conditions) • Solubility: ↑ to ∼90% (severe: pH 12, 120 °C)	Effective for solubility enhancement, especially under mild conditions.	Harsh conditions induce side reactions (racemization, lysinoalanine); precise control needed.	Protein ingredients where high solubility at neutral/alkaline pH is the primary goal.	Guan et al. (2017)
	Phosphorylation (with STMP at pH 9)	Covalent attachment of phosphate groups to Ser/Thr/Tyr residues, increasing negative charge and hydrophilicity.	• Solubility: ↑ from 6.67% to 58.4% • EAI: ↑ from 1.70 to 13.72 m^2^/g • Secondary structure: β-sheet ↓; α-helix/β-turn ↑	Extremely effective at improving solubility near the isoelectric point; uses GRAS reagents.	Chemical modification label; potential regulatory and consumer perception hurdles.	Neutral-pH fortified foods, beverages, and emulsion systems where solubility is limiting.	Hu et al. (2019); Ma et al. (2017)
Biological	Limited Enzymatic Hydrolysis (DH < 10%, e.g., Trypsin)	Selective cleavage of peptide bonds increases ionizable termini, reduces molecular weight, and enhances flexibility.	• Solubility: ↑ • Molecular flexibility: ↑ • High-MW aggregates: ↓	High specificity and mild conditions; clean-label; improves digestibility.	Enzyme cost; requires precise control of DH to avoid bitterness (DH>10%).	Clinical/sports nutrition liquids, bioactive peptides, easily digestible protein ingredients.	Zang et al. (2019); Singh et al. (2021); Wang et al. (2025)
	Transglutaminase Cross-linking (0.5 kat/kg, 3 h)	Catalyzes isopeptide bond formation between Gln and Lys residues, creating a cross-linked protein network.	• Foam capacity: ↑ to 375% • Foam volume ratio: 79.45% • Gel network: more homogeneous and compact	Builds strong, viscoelastic gel structures; improves foam stability; food-grade enzyme.	Can reduce protein solubility; relatively high enzyme cost; reaction times can be long.	Plant-based cheese, yogurt, surimi analogues, and emulsion gels requiring firm texture.	Wu et al., 2023; Liu et al. (2024)
	Fermentation (Yeast/Natural)	Microbial proteases hydrolyze proteins; metabolic activity modifies composition and structure *in situ*.	• Protein content: ↑ (YFRBPC: 72.50%) • Total amino acids: ↑ (YFRBPC: 123.16 • DPPH inhibition: ↑ 58.62% • Td: ↑ 108.01 °C • Solubility at alkaline pH: ↑	Can improve flavor and nutritional profile; environmentally friendly; low-tech infrastructure.	Process variability; long fermentation time; challenges in ensuring batch-to-batch consistency.	Bakery products, fermented functional ingredients, upgrading animal feed protein quality.	Chinma et al. (2014)

Note: EAI: emulsifying activity index; ESI: emulsifying stability index; RBG: rice bran globulin; YFRBPC: yeast-fermented rice bran protein concentrate; DH: degree of hydrolysis; Td: denaturation temperature.

Analysis from the perspective of functional enhancement shows clear application-specific strengths. For the core challenge of improving RBP solubility, HHP and limited enzymatic hydrolysis are particularly effective, with HHP treatment at 200 MPa for 40 min reported to increase solubility by 76.69% (Yi and Liu, 2023). For developing strong gel textures, transglutaminase cross-linking and extrusion are superior choices; the former enables the formation of homogeneous, dense three-dimensional networks suitable for plant-based cheese analogues (Liu et al., 2024). In enhancing emulsion stability, glycosylation via the Maillard reaction is highly effective, increasing the emulsifying stability index of rice bran globulin from 50.66% to 65.37% (Zhang et al., 2024). In comparison, physical methods such as steam flash explosion and ultrasound also improve solubility and emulsification, though generally to a more moderate extent than chemical approaches.

The selection of a modification technology must extend beyond functional performance to include practical considerations of process feasibility, cost, and labeling requirements. As outlined in Table 1, physical methods generally offer clean-label advantages, yet HHP involves high capital investment, and ultrasound faces challenges in continuous large-scale operation. Chemical methods can deliver significant functional enhancements, but glycosylation requires precise reaction control to prevent advanced Maillard products, and modifications like phosphorylation involve chemical reagents that may raise regulatory or consumer perception concerns. Biological methods operate under mild conditions with high specificity, but their industrial adoption is influenced by enzyme cost, the need for precise control of hydrolysis degree to avoid bitterness, and batch consistency in fermentation processes.

Consequently, a logical selection framework emerges for specific applications. For developing high-solubility protein beverages or clinical nutrition products, HHP or controlled enzymatic hydrolysis should be prioritized. For food systems requiring long-term emulsion stability, such as dressings or creams, glycosylation represents the most effective strategy, with microfluidization or ultrasound serving as clean-label physical alternatives. For products demanding strong, elastic gel structures like meat or cheese analogues, transglutaminase cross-linking or extrusion are the most applicable technologies. In cost-sensitive applications where a clean label is paramount, thermal treatment, fermentation, or ultrasound may be preferred, albeit with an acceptance of their respective functional ceilings. In summary, there is no universal optimal modification method for RBP. Its successful application requires a strategic balance between the target functionality, production economics, process scalability, and market-driven labeling constraints.

## 6 Conclusion and future trends

**Fig. 4:**
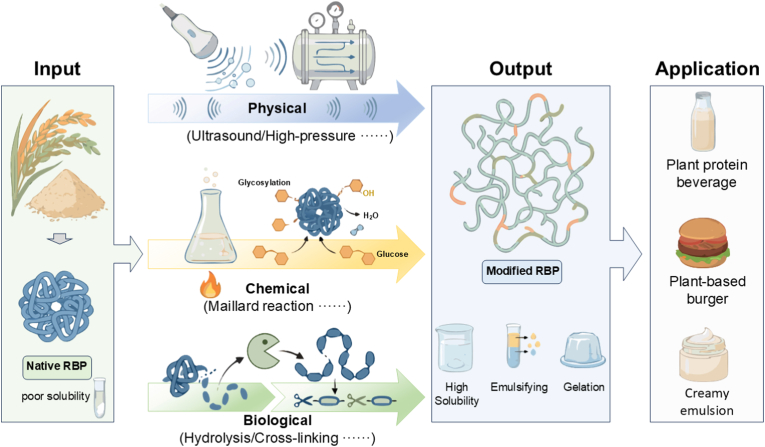
The different modification methods and applications of rice bran protein.

This review consolidates the current scientific landscape of modifying RBP, a high-quality yet functionally limited plant protein. As shown in Fig. 4, through a critical examination of physical, chemical, and biological strategies, this review has established how distinct mechanisms, ranging from mechanical disaggregation and thermal unfolding to covalent conjugation and enzymatic tailoring, can be strategically employed to overcome RBP's inherent deficiencies. The resulting enhancements in solubility, emulsification, gelation, and interfacial stability, as quantified throughout this work, demonstrate the considerable potential of modified RBP as a versatile food ingredient. More importantly, the synthesis presented here moves beyond a descriptive catalog by introducing a structured, comparative framework (Section 4, Table 1). This framework explicitly links modification principles to functional outcomes and practical application scenarios, while providing a clear assessment of technological trade-offs related to scalability, cost, and clean-label status.

To translate this potential into widespread industrial adoption, future research must address several pivotal challenges. First, the exploration of synergistic hybrid processes, which intelligently combine physical, chemical, and biological treatments, could unlock superior functionality while mitigating the limitations of individual methods. Second, a deeper understanding of modified RBP's performance within complex, multi-component food matrices is essential. Fundamental studies on its interactions with other macromolecules (e.g., polysaccharides, lipids) under realistic processing and storage conditions will be critical for predictable product formulation. Finally, focused efforts on process intensification, energy optimization, and economic validation are urgently needed to scale the most promising laboratory successes into commercially viable, sustainable unit operations.

In summary, the targeted modification of RBP represents a significant opportunity for the sustainable valorization of a major agricultural by-product. By providing a mechanistic foundation, a critical comparative analysis, and a roadmap for future development, this review aims to guide the rational design of RBP-based ingredients. Advancing this field will not only enhance the functionality of plant proteins but also contribute to building more efficient and innovative food systems.

## Authorship contribution

Jiao-jiao Yin: Writing-review & editing, Funding acquisition. Yuan Zou: Writing-original draft. Kai Yao: Investigation. Pao Gao: Conceptualization. Wu zhong: Conceptualization. Xing-he Zhang: Conceptualization. Li Wang: Writing-review & editing.

## Declaration of competing interest

No.
